# Supplementary material for: DynamicBind: predicting ligand-specific protein-ligand complex structure with a deep equivariant generative model
Source: Nat Commun. 2024 Feb 5;15:1071. doi: 10.1038/s41467-024-45461-2 (PMC10844226; doi:10.1038/s41467-024-45461-2)
Supplement: Supplementary file 1 — Supplementary Information [file 41467_2024_45461_MOESM1_ESM.pdf]

Supplementary information for DynamicBind: Predicting ligand-specific protein-ligand complex structure with a deep equivariant generative model

January 24, 2024

Model	RMSE(↓)	Pearson(↑)	Spearman(↑)	MAE(↓)
GIGN	1.736	0.286	0.318	1.330
TransformerCPI	1.643	0.470	0.480	1.317
MONN	1.371	0.545	0.535	1.103
TankBind	1.436	0.597	0.610	1.119
DynamicBind(1)	1.301	0.665	0.634	1.060
DynamicBind(5)	1.269	0.681	0.642	1.024
DynamicBind(10)	1.256	0.687	0.656	1.016
DynamicBind(20)	1.253	0.689	0.656	1.013
DynamicBind(40)	1.253	0.689	0.655	1.013

Supplementary Table 1: **Binding affinity benchmark on the PDBbind test set.** DynamicBind has achieved state-of-the-art performance in binding affinity prediction on the PDBbind test set. We also notice that, as the number of samples increases from 1 to 20, the ensemble average of predicted affinity has lower RMSE and higher Pearson and Spearman correlation with the ground truth.

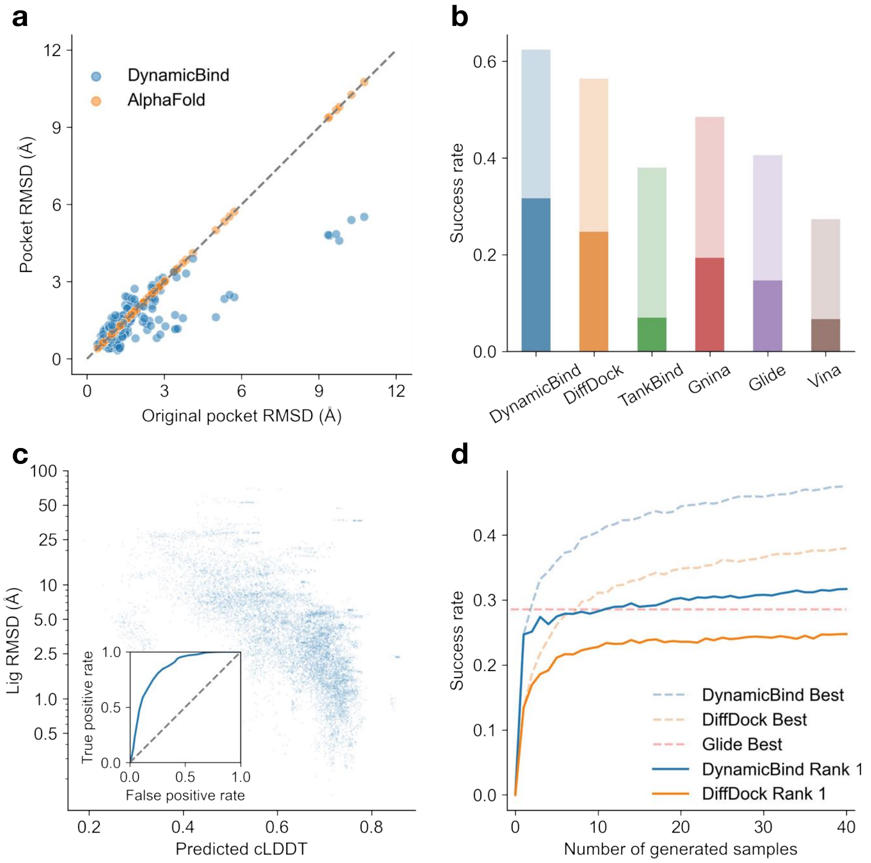

Supplementary Figure 1: **Benchmark results for PDBbind.** **a**, the protein structures predicted by DynamicBind have significantly lower pocket RMSD in comparison with the input AlphaFold structures. **b**, the success rate of DynamicBind, 0.317, is 25% higher than the second best method (DiffDock, 0.247) under stringent criteria. **c**, the area under curve for plot of the true positive rate vs false positive rate under RMSD 2Å threshold is 0.85. The success rate of DynamicBind sees a sharp increase initially as the number of generated samples rises. **d**, this growth begins to plateau for rank 1 results once the sample count surpasses 20, yet it continues to ascend for the best sampled structures.

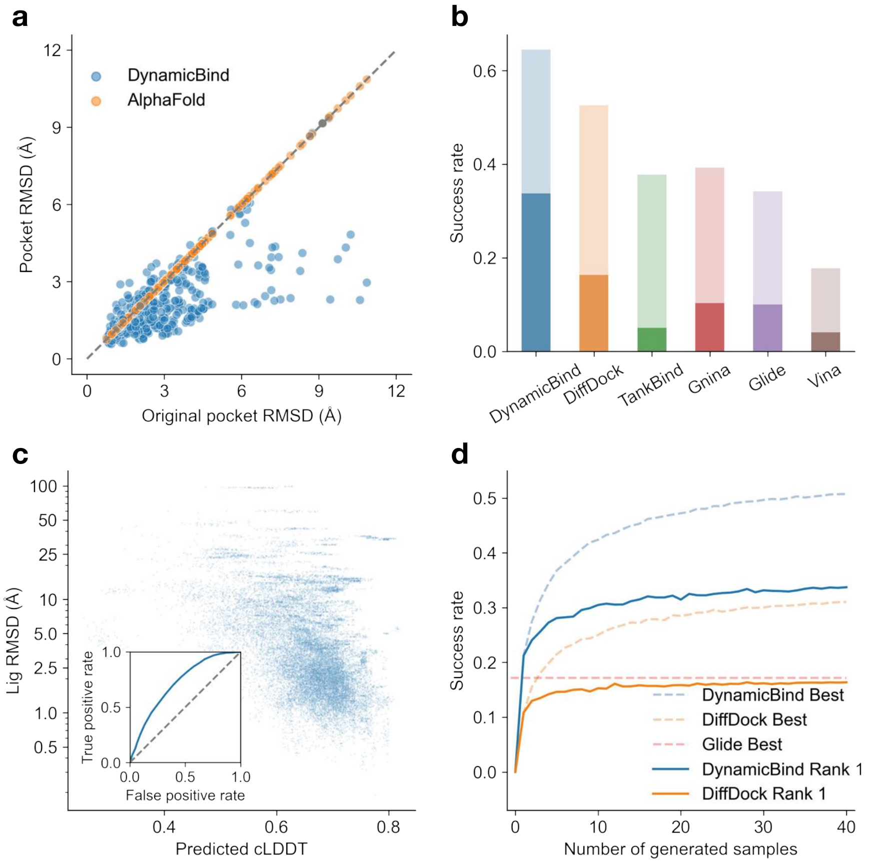

Supplementary Figure 2: **Benchmark results for MDT test set.** **a**, the protein structures predicted by DynamicBind have significantly lower pocket RMSD in comparison with the input AlphaFold structures. **b**, the success rate of DynamicBind, 0.337, is about 2 times the second best method (DiffDock, 0.163) under stringent criteria. **c**, the area under curve for plot of the true positive rate vs false positive rate under RMSD 2Å threshold is 0.71. The success rate of DynamicBind sees a sharp increase initially as the number of generated samples rises. **d**, this growth begins to plateau for rank 1 results once the sample count surpasses 20, yet it continues to ascend for the best sampled structures.

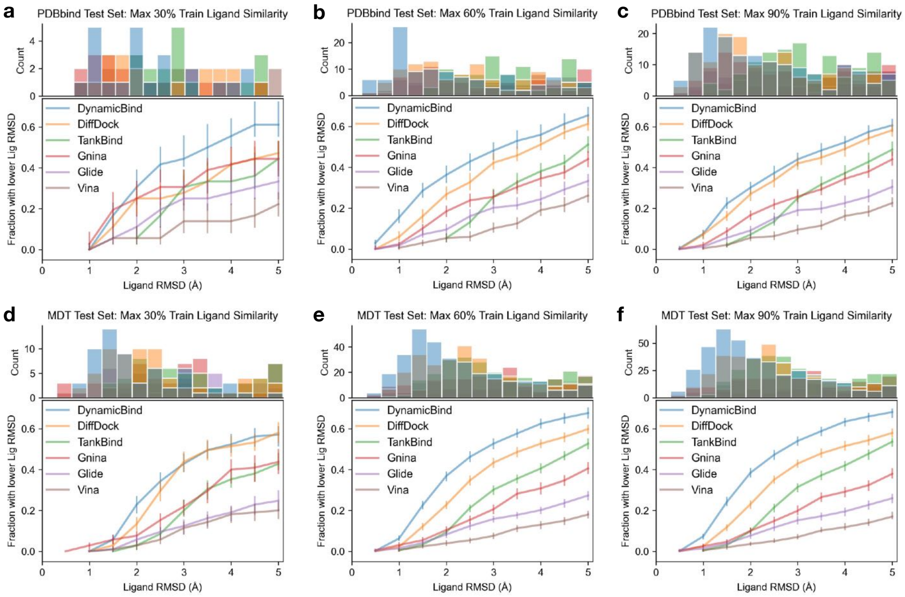

Supplementary Figure 3: **Benchmark results with bootstrapped confidence interval for PDBbind and MDT test set filtered at different maximum ligand similarity to the training set.** The ligand similarity is calculated by Morgan fingerprint Tanimoto Distance using RDKit [1]. For the PDBBind test set, 11.8%, 55.4%, and 83.1% of ligands have a maximum similarity less than 0.3, 0.6, and 0.9 to the training set, respectively. On the other hand, for the MDT test set, the percentages are 17.5%, 76.9%, and 93.3% for the respective similarity thresholds (0.3, 0.6, and 0.9). DynamicBind consistently outperforms other methods across different ligand similarity threshold for both test sets. For each method, we resample 100 times with replacement, and plot 68% confidence intervals.

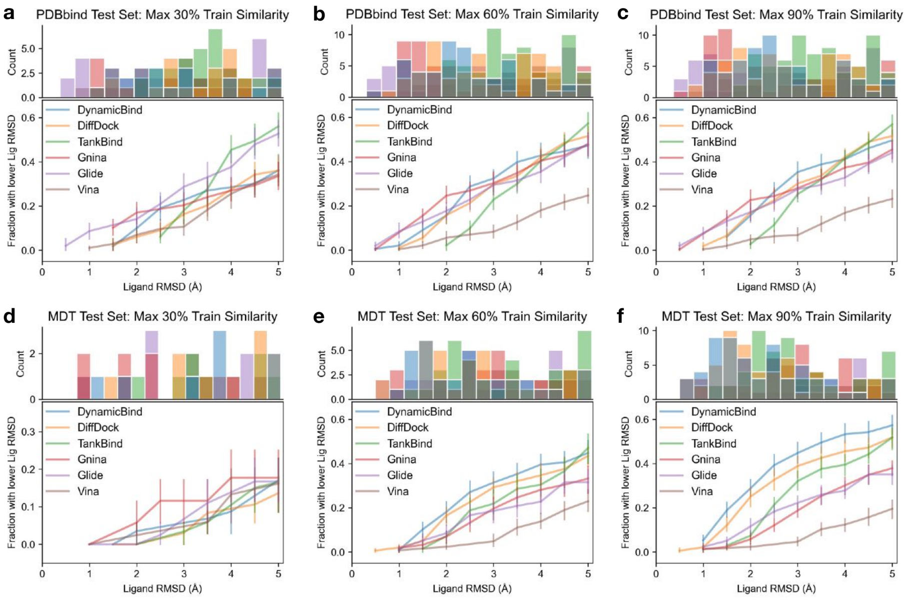

Supplementary Figure 4: **Benchmark results with bootstrapped confidence interval for PDBbind and MDT test set filtered at different maximum protein sequence similarity to the training set.** The sequence similarities between training and test set proteins were assessed utilizing the Smith–Waterman algorithm [2]. While there was an observed decrease in model accuracy, it is encouraging that DynamicBind outperformed the state-of-the-art deep learning method, DiffDock, at the stringent 30% similarity threshold for the PDBbind test set. For traditional force-field-based methods like Glide, Vina, and Gnina, we performed local docking due to their limited effectiveness in global docking scenarios. The box center for these dockings was set at the center of mass of the native ligand and the box size is extend 4Å buffer on all six sides around the native ligand. This approach inherently benefits these traditional methods, as opposed to deep learning-based methods, which perform global docking without prior knowledge of the pocket location. For each method, we resample 100 times with replacement, and plot 68% confidence intervals.

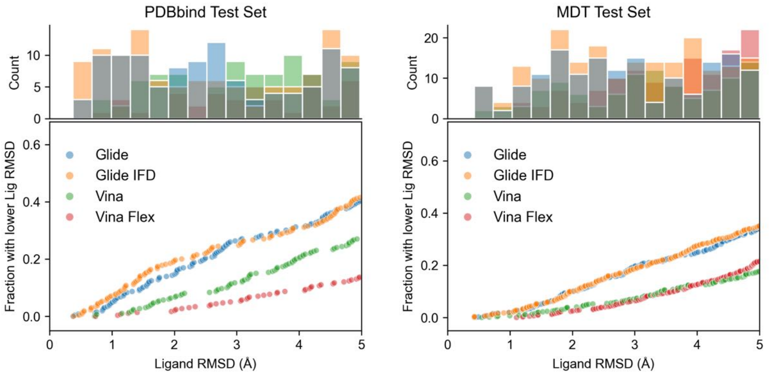

Supplementary Figure 5: **Additional benchmark results comparing Glide Induced Fit Docking (IFD) and Vina Flex with their regular counterparts.** Glide IFD exhibits a slight improvement over regular Glide, while Vina Flex has a marginally lower performance compared to standard Vina. However, both Glide IFD and Vina Flex are broadly similar in efficacy to their respective standard versions. It's noteworthy that both Glide IFD and Vina Flex can only slightly modify the protein backbone's location, which may not be sufficient to accommodate the ligand effectively.

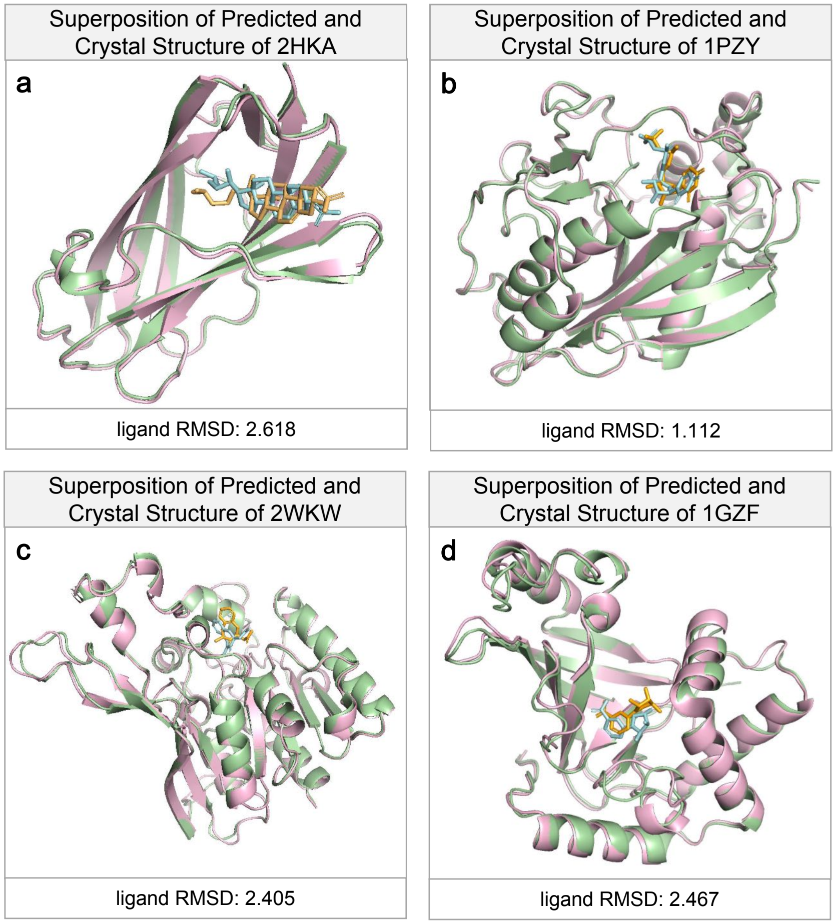

Supplementary Figure 6: **Prediction result for Cryptosite [3] test cases with maximum sequence similarity to the training set below 30%.** Ligand RMSD value are around 2Å, suggesting accurate identification of cryptic sites by our model. The crystal structures are depicted with proteins in pink and ligands in cyan. Predictions from our model are illustrated with proteins in green and ligands in orange.

Hyperparameters.

**Hyperparameter**	**Value**
convolution layers	6
number of scalar features	72
number of vector features	12
Learning rate	0.001
Number of epochs	400
Batch size	40
Betas	(0.9, 0.999)
Weight decay	0.0
Dropout rate	0.1
σ_{tr_min}	0.1
σ_{tr_max}	19
σ_{rot_min}	0.03
σ_{rot_max}	1.55
σ_{tor_min}	0.0314
σ_{tor_max}	3.14
σ^t	$\sigma_{min}^{1-t}\sigma_{max}^t$

Supplementary Table 2: Hyperparameters of the model

Supplementary Note 1 Algorithm 1 Training procedure of DynamicBind

Require: $\mathbf{x}^{gt}$, $\mathbf{x}^{holo}$, $\mathbf{x}^{apo}$, RDKit conformation $\mathbf{c}$, Binding affinity

1: Match $\mathbf{x}_0^l \leftarrow \text{argminRMSD}(\mathbf{c}, \mathbf{x}^{gt})$

2: Compute $w_i \leftarrow \exp\big(\text{ReLU}(\frac{\|\mathbf{x}_i^p - \overline{\mathbf{x}}_0^l\|^2 - 6}{10})\big)$

3: For each residue, compute:

$$\mathbf{tr}_i^*, \mathbf{rot}_i^* \leftarrow \text{Kabsch}(\mathbf{x}_{i,(N,C_\alpha,C)}^{holo} - \mathbf{x}_{i,C_\alpha}^{holo}, \mathbf{x}_{i,(N,C_\alpha,C)}^{apo} - \mathbf{x}_{i,C_\alpha}^{holo})$$

4: Sample $t \sim \text{Uni}([0,1])$

5: Sample $\boldsymbol{\Delta}\mathbf{tr}^l, \boldsymbol{\Delta}\mathbf{rot}^l, \Delta T_k^l, \boldsymbol{\Delta}\mathbf{tr}_i^p, \boldsymbol{\Delta}\mathbf{rot}_i^p, \Delta T_{i,k}^p$

6: Set $\Delta R^l \leftarrow$ Rotation matrix of $\boldsymbol{\Delta}\mathbf{rot}^l$

7: Set $\Delta R_i^p \leftarrow$ Rotation matrix of $\boldsymbol{\Delta}\mathbf{rot}_i^p$

8: Set $\mathbf{x}_t^l \leftarrow (\Delta T_0^l \circ \cdots \Delta T_k^l)(\Delta R^l(\mathbf{x}_0^l - \overline{\mathbf{x}}_0^l) + \overline{\mathbf{x}}_0^l + \boldsymbol{\Delta}\mathbf{tr}^l)$

9: Set $\mathbf{x}_t^p \leftarrow (\Delta T_{i,0}^p \circ \cdots \Delta T_{i,k}^p)(\Delta R_i^p(\mathbf{x}_i^{holo} - \mathbf{x}_{i,C_\alpha}^{holo}) + \mathbf{x}_{i,C_\alpha}^{holo} + \boldsymbol{\Delta}\mathbf{tr}_i^p)$

10: Compute cLDDT $\leftarrow$ perturbed contact LDDT

11: Predict $\mathbf{tr}^l, \mathbf{rot}^l, T^l, \mathbf{tr}_i^p, \mathbf{rot}_i^p, T_i^p, A, D = \text{model}(\mathbf{x}_t^l, \mathbf{x}_t^p, t)$

12: Compute:

$$\boldsymbol{\Delta}\tilde{\mathbf{rot}}^l \leftarrow \text{same opposite rotation vector of } \boldsymbol{\Delta}\mathbf{rot}^l$$
$$\boldsymbol{\Delta}\tilde{\mathbf{rot}}_i^p \leftarrow \text{same opposite rotation vector of } \boldsymbol{\Delta}\mathbf{rot}_i^p$$

13: Compute gradients and take optimization:

$$\mathcal{L}_1 = \|\mathbf{tr}^l - \boldsymbol{\Delta}\mathbf{tr}^l\|^2$$
$$\mathcal{L}_2 = \min(\|\mathbf{rot}^l - \boldsymbol{\Delta}\mathbf{rot}^l\|^2, \|\mathbf{rot}^l - \boldsymbol{\Delta}\tilde{\mathbf{rot}}^l\|^2)$$
$$\mathcal{L}_3 = \sum_{i=1}^{m(l)} \frac{1 - \cos\big(T_k^l - \Delta T_k^l\big)}{m(l)}$$
$$\mathcal{L}_4 = \frac{\sum_{i=1}^{n(p)} w_i \|\mathbf{tr}_i^p - \boldsymbol{\Delta}\mathbf{tr}_i^p\|^2}{\sum_{i=1}^{n(p)} w_i}$$
$$\mathcal{L}_5 = \frac{\sum_{i=1}^{n(p)} w_i (\min(\|\mathbf{rot}_i^p - \boldsymbol{\Delta}\mathbf{rot}_i^p\|^2, \|\mathbf{rot}_i^p - \boldsymbol{\Delta}\tilde{\mathbf{rot}}_i^p\|^2))}{\sum_{i=1}^{n(p)} w_i}$$
$$\mathcal{L}_6 = \frac{\sum_{i=1}^{n(p)} w_i \Big(\sum_{i=1}^{m(p)} \frac{1 - \cos\big(T_k^p - \Delta T_k^p\big)}{m(p)}\Big)}{\sum_{i=1}^{n(p)} w_i}$$
$$\mathcal{L}_7 = \|A - \text{Affinity}\|^2, \ \mathcal{L}_8 = \|D - \text{cLDDT}\|^2$$
$$\mathcal{L} = \sum_{i=1}^{8} \lambda_i \mathcal{L}_i$$

Supplementary Note 2 Algorithm 2 Inference procedure of DynamicBind

Require: RDKit conformation $\mathbf{c}$, $\mathbf{x}^{apo}$, Total steps N

Sample $\Delta\mathbf{tr}^l, \Delta\mathbf{rot}^l, \Delta T_k^l$
Set $\Delta R^l \leftarrow$ Rotation matrix of $\Delta\mathbf{rot}^l$
Set $\mathbf{x}_N^l \leftarrow (\Delta T_0^l \circ \cdots \Delta T_k^l)(\Delta R^l(\mathbf{c} - \overline{\mathbf{c}}) + \overline{\mathbf{c}} + \Delta\mathbf{tr}^l)$
Set $\mathbf{x}_N^p \leftarrow \mathbf{x}^{apo}$
Set $n = N$
while $n > 0$ **do**
  Set $t = n/N$
  Set $\Delta(\sigma_{\mathbf{tr}}^l)^2 \leftarrow \frac{1}{N}(\sigma_{\mathbf{tr}}^l)^2$, $\Delta(\sigma_{\mathbf{rot}}^l)^2 \leftarrow \frac{1}{N}(\sigma_{\mathbf{rot}}^l)^2$, $\Delta(\sigma_{tor}^l)^2 \leftarrow \frac{1}{N}(\sigma_{tor}^l)^2$
  Predict $\mathbf{tr}^l, \mathbf{rot}^l, T^l, \mathbf{tr}_i^p, \mathbf{rot}_i^p, T_i^p \leftarrow \text{model}(\mathbf{x}_t^l, \mathbf{x}_t^p, t)$
  if $n > 1$ **then**
    Sample:
      $\mathbf{z}_{\mathbf{tr}}^l \sim \mathcal{N}\left(0, \Delta(\sigma_{\mathbf{tr}}^l)^2\right)$
      $\mathbf{z}_{\mathbf{rot}}^l \sim \mathcal{N}\left(0, \Delta(\sigma_{\mathbf{rot}}^l)^2\right)$
      $\mathbf{z}_{tor}^l \sim \mathcal{N}\left(0, \Delta(\sigma_{tor}^l)^2\right)$
    Set $\mathbf{tr}^l \leftarrow \mathbf{tr}^l + \mathbf{z}_{\mathbf{tr}}^l$
    Set $\mathbf{rot}^l \leftarrow \mathbf{rot}^l + \mathbf{z}_{\mathbf{rot}}^l$
    Set $T^l \leftarrow T^l + \mathbf{z}_{tor}^l$
  end if
  Set $R^l \leftarrow$ Rotation matrix of $\mathbf{rot}^l$
  Set $R_i^p \leftarrow$ Rotation matrix of $\mathbf{rot}_i^p$
  Set $\mathbf{x}_{n-1}^l \leftarrow \text{RMSDAlign}((T_n^l \circ \cdots T_k^l)\mathbf{x}_n^l, R^l(\mathbf{x}_n^l - \overline{\mathbf{x}}_n^l) + \overline{\mathbf{x}}_n^l + \mathbf{tr}^l)$
  Set $\mathbf{x}_{n-1}^p \leftarrow (T_{i,0}^p \circ \cdots T_{i,k}^p)(R_i^p(\mathbf{x}_{i,n}^p - \mathbf{x}_{i,n,C_\alpha}^p) + \mathbf{x}_{i,n,C_\alpha}^p + \mathbf{tr}_i^p)$
  Set $n = n - 1$
end while
Predict $A, D \leftarrow \text{model}(\mathbf{x}_0^l, \mathbf{x}_0^p, t')$
Return $\mathbf{x}_0^l$, $\mathbf{x}_0^p$, A, D

References

1. Landrum, G. *et al. RDKit: A software suite for cheminformatics, computational chemistry, and predictive modeling* 2013.
2. Zhao, M., Lee, W.-P., Garrison, E. P. & Marth, G. T. SSW library: an SIMD Smith-Waterman C/C++ library for use in genomic applications. *PloS one* **8,** e82138 (2013).
3. Cimermancic, P. *et al.* CryptoSite: expanding the druggable proteome by characterization and prediction of cryptic binding sites. *Journal of molecular biology* **428,** 709–719 (2016).
